# Editorial: Innovative strategies and advancements in phototherapy for enhanced cancer treatment

**DOI:** 10.3389/fmed.2026.1789533

**Published:** 2026-02-10

**Authors:** Leonardo Bianchi, Leopoldo Sitia, Gabriela Paroni, Sanjeev Soni

**Affiliations:** 1 Department of Mechanical Engineering, Politecnico di Milano, Milan, Italy; 2 Tumor Biology and Vascular Targeting Unit, Comprehensive Cancer Center, IRCCS San Raffaele Scientific Institute, Milan, Italy; 3 Department of Biochemistry and Molecular Pharmacology, Istituto di Ricerche Farmacologiche Mario Negri IRCCS, Milan, Italy; 4 Bio-medical Instrumentation Department, CSIR-Central Scientific Instruments Organization, Chandigarh, India; 5 Academy of Scientific and Innovative Research (AcSIR), Ghaziabad, India

**Keywords:** nanomedicine, nanoparticle-based phototherapy, photodynamic therapy, photoimmunotherapy, photomedicine, phototherapy, photothermal therapy, thermal ablation

## Introduction

Light-triggered anticancer therapies are increasingly explored as minimally invasive interventions that can confine damage to the illuminated region while preserving surrounding tissues and function. In phototherapy, externally delivered light activates or drives therapeutic effects through photoresponsive agents or materials, enabling spatiotemporal control. Depending on the agent and context, phototherapy can induce direct tumor cell injury, vascular disruption, and downstream immune effects, while offering opportunities for repeatability and procedure-based integration.

Nevertheless, phototherapy is still not broadly adopted as first-line care across most tumor types. Key barriers include selective accumulation of photoactive agents at the tumor site, microenvironmental constraints (notably oxygen availability for oxygen-dependent photochemical reactions), light penetration depth, and the practical challenge of delivering and standardizing light dose and dosimetry across heterogeneous lesions and anatomical settings. The studies highlighted in this Research Topic reflect a translational continuum spanning from focal, organ-preserving concepts to minimally invasive procedural delivery and post-operative adjuvant feasibility, as well as approaches that consider phototherapy-induced antitumor immune effects in treatment design and outcome assessment, and the enabling role of nanomedicine.

## Phototherapy approaches: from precision local control to multimodal, immune-modulatory, and nanomedicine-enabled oncologic strategies

A disease-focused foundation is provided by Xu et al., who review photodynamic therapy (PDT) for localized prostate cancer. They frame the clinical motivation in terms of balancing tumor control with functional outcomes. Mechanistically, they describe PDT as the interplay of light, tissue oxygen, and a photosensitizer (PS), where photoexcitation generates reactive oxygen species via two mechanisms, including singlet oxygen formation, enabling spatially confined tumor ablation. Beyond mechanism, the review emphasizes that delivery remains a central determinant of success and highlights nanotechnology-enabled PS formulations as a route to improve tumor selectivity and performance, aligning focal precision with practical translational needs.

A clinically distinct, procedure-integrated translation is presented by Mai et al. in an exploratory pilot study of medical thoracoscopic PDT for metastatic pleural tumors with malignant pleural effusion in non-small cell lung cancer. Their approach links phototherapy to endoscopic workflow and lesion morphology: plaque-like lesions received PDT alone (energy density of 384 J/cm^2^), while nodular-like and mass-like lesions were treated with argon plasma coagulation (APC) followed by PDT at 480 and 576 J/cm^2^, respectively. The authors report an overall response rate of 82.1% at week 6 and 89.3% at week 12; time to progression was longer in the PDT ± APC group than in the APC group from their previous study (median 20.7 vs. 14.2 months; P = 0.006; hazard ratio 0.35), with a 1-year survival rate of 75% (21/28), and the toxicities related to treatment were notably minor.

Wang et al. add a complementary perspective through a case report of perianal Paget's disease managed with adjuvant PDT after wide local excision and flap reconstruction. Adjuvant PDT was initiated 2 months after surgery and was repeated every 2 weeks for a total of three sessions. The reported protocol used methyl aminolevulinate (applied around the lesion with a margin), an incubation period, and red-light irradiation at 620 nm (37 J/cm^2^) for 10 min, with only mild pain at the irradiated site managed conservatively. While inherently limited by single-patient generalizability, the case highlights a realistic use scenario for phototherapy: post-operative local management in anatomically sensitive regions where function preservation and tolerability are central to decision-making.

Shifting the focus to thermal mechanisms and immunity, Chen et al. review photothermal therapy (PTT) in breast cancer with an emphasis on immune modulation. PTT relies on photothermal agents that convert absorbed light into heat, producing local tumor injury. Importantly, the review frames PTT as more than ablation, noting that heat-induced tumor cell death can release antigens and promote antigen presentation, engaging both innate and adaptive immune responses. The authors also discuss constraints that can limit immune benefit, including insufficient antigen release and immunosuppressive tumor microenvironments, reinforcing that incorporating immune effects into treatment design is essential if photothermal approaches are to contribute beyond local cytoreduction.

At a broader level, Tian and Chen provide a global view through a bibliometric and visualization analysis of nanomedicine research in colorectal cancer (2011–2024), based on 3,105 Web of Science Core Collection publications. Their mapping identifies nanoparticles and nanomedicine-based drug delivery systems as major hotspots and highlights PDT, immunogenic cell death, and tumor microenvironment among frontier themes. Although not a disease-specific phototherapy article, this analysis reinforces a cross-cutting translational point: clinical success often depends on effective delivery, tumor-specific targeting, and microenvironmental considerations, areas in which nanomedicine can play an enabling role.

## Future perspectives

Recent syntheses frame cancer phototherapy as an evolving therapeutic paradigm that includes PDT, PTT, and photoimmunotherapy, while emphasizing that clinical translation is increasingly governed by delivery, dosimetry, and standardized workflows (1). A parallel argument is that mechanism-led development is essential, particularly for nano-enabled platforms where uncertain modes of action can impede standardization and clinical translation (2). Perspective work also stresses the importance of aligning promise with rigorous clinical evaluation and realistic constraints of light delivery for superficial and deep-seated tumors (3). In addition, combination strategies are increasingly framed as a pragmatic route to overcome single-modality limitations, provided that added complexity is justified by measurable gains in efficacy, safety, or durability (4).

Looking forward, the field will benefit from tighter coupling between light-delivery engineering and clinical workflows, delivery systems that are explicitly designed around tumor microenvironment constraints, and the incorporation of immune-related outcome measures that capture effects beyond local ablation (1, 4).

Within this landscape, thematic Research Topics like the present one can play a concrete role by consolidating procedure designs, reporting parameters transparently, and connecting mechanistic insights to clinically implementable workflows.

## Conclusion

The present Research Topic connects focal PDT concepts in localized prostate cancer with procedure-integrated intrapleural PDT in metastatic pleural disease, illustrates feasible adjuvant PDT implementation in a rare and anatomically sensitive indication, and highlights immune-modulatory PTT frameworks alongside the enabling role of nanomedicine. Together, these contributions highlight both the opportunities and the key challenges that will need careful attention as phototherapy moves beyond selectively applied interventions toward combination therapies and broader clinical relevance, helping to inform more standardized and evidence-driven implementation in clinical practice.
